# Transcranial direct current stimulation (tDCS) targeting the postcentral gyrus reduces malevolent creative ideation

**DOI:** 10.1093/scan/nsad019

**Published:** 2023-03-24

**Authors:** Zhenni Gao, Kelong Lu, Ning Hao

**Affiliations:** Institute of Brain and Psychological Sciences, Sichuan Normal University, Chengdu 610066, China; School of Mental Health, Wenzhou Medical University, Wenzhou 325035, China; Shanghai Key Laboratory of Mental Health and Psychological Crisis Intervention, School of Psychology and Cognitive Science, East China Normal University, Shanghai 200062, China

**Keywords:** malevolent creativity, tDCS, postcentral gyrus, emotional recognition

## Abstract

Malevolent creativity (MC) is defined as a manifestation in which people propose to materially, mentally or physically harm themselves or others in a novel manner. Malevolent creative ideation can be inhibited by high moral emotions (i.e. sympathy, guilt and shame) and low negative emotions, which promote prosocial behaviors. Given that the right postcentral gyrus (PCG) is involved in generating sympathy and emotional recognition for others and the right middle frontal gyrus (MFG) is involved in emotional regulation, we suggest that the right PCG and right MFG may play important roles in malevolent creative ideation. In Study 1, we recruited 98 healthy and right-handed college participants (80 females, age = 21.11 ± 2.00 years) and examined the role of the right PCG in malevolent creative ideation using transcranial direct current stimulation (tDCS). The results showed that the accuracy of emotional recognition changed when the right PCG received electrical stimulation. Enhancing the activation of the right PCG reduced MC originality and fluency, whereas inhibiting it increased MC originality and fluency. In Study 2, we recruited 91 healthy and right-handed college participants (74 females, age = 21.22 ± 2.28 years) and examined the role of the right MFG in malevolent creative ideation using tDCS. The results showed no significant difference in malevolent creative performance between the pre- and post-test when electrical stimulation was applied over the right MFG. These findings indicate that enhancing the activation of the right PCG, which is closely correlated with emotional recognition, reduces an individual’s malevolent creative ideation.

## Introduction

Creativity is considered as the ability to generate novel and useful ideas or products (Runco and Jaeger, 2012). As one of the dark aspects of creativity, malevolent creativity (MC) is defined as a manifestation in which individuals propose to materially, mentally or physically harm themselves or others in a novel manner (Cropley *et al.*, 2008; Cropley, 2010). Based on the definition of MC, it involves not only regular creativity but also a purpose to harm others mentally or physically. This emphasizes that MC is a more complex cognition and intrinsically differs from regular creativity. While MC may be deemed essential for a particular group or individual to fulfill their goals, it has negative consequences for other groups or individuals (Cropley *et al.*, 2008). Examples of MC exist everywhere, such as fraud and creative crimes, and can lead to severe negative consequences. Research focused on the relationship between malevolent creative performance and criminal behavior (e.g. murder, abuse and violence), personality, motivation and moral emotion (Cropley *et al.*, 2008; Harris *et al.*, 2013; Hao *et al.*, 2020; Cheng *et al.*, 2021b). A possible link between MC and criminal behavior can be found when individuals break the law. Cropley *et al.* (2008) demonstrated that a creative criminal might carry out a crime in an original way. Personality traits such as aggression are correlated with MC. Individuals who show higher physical aggression exhibit higher levels of MC (Lee and Dow, 2011). Hao *et al.* (2020) investigated the relationship between motivation and MC and found that approach motivation predicted MC, while avoidance motivation was negatively associated with MC.

Moral emotion and MC may be closely linked. Kapoor and Kaufman (2022) proposed the AMORAL model in relation to MC. The AMORAL model consists of the Antecedents, Mechanisms (individual), Operants (environmental), Realization, Aftereffects, and Legacy of dark creativity. Regarding mechanisms (individual), these tend to include certain individual-level features that can inhibit or promote MC. In particular, this individual-level feature refers to a range of social and emotional abilities, such as identifying and managing emotions (Kapoor and Kaufman, 2022).

On the one hand, identifying emotions is closely associated with malevolent creative performance. Experiencing moral emotions, such as guilt and shame based on identifying emotions, can discourage creative individuals from engaging in more malevolent actions (Haidt, 2003b; Tangney *et al.*, 2007). Guilty conscience, viewed as a superego response to unacceptable impulses (Babcock, 1983), can promote prosocial behaviors (Ferguson and Branscombe, 2010; Rees *et al.*, 2015; Hurst and Sintov, 2022). Shame also leads to higher levels of guilt, which in turn promotes positive behavior (Rees *et al.*, 2015). In addition, sympathy, another moral emotion, and MC may be closely related. Sympathy is defined as an understanding of others’ situations and feelings (Eisenberg, 2000; Malti *et al.*, 2013). Sympathy and prosocial/cooperative behavior are generally found to have a positive relationship (Eisenberg and Miller, 1987; Eisenberg *et al.*, 1989; Carlo *et al.*, 2010; Grueneisen and Warneken, 2022). Sympathy can motivate individuals to react sympathetically to others’ suffering, and individuals with higher sympathy tend to find novel and prosocial solutions that alleviate suffering and promote well-being (Yang and Yang, 2016), which is the opposite of MC. Unethical behavior is significantly predicted by MC (Gino and Ariely, 2012). When implementing malevolent creative ideas, individuals may not necessarily gain substantial profits, especially when deceiving and retaliating against others. In this case, individuals gain psychological satisfaction by putting others in an unfortunate situation or causing them to feel negative emotions. This suggests that individuals with high levels of MC could predict their actions that will lead to a negative emotional reaction in the recipient. However, they may become less emotionally activated by such actions in order to avoid sympathy, guilt and shame.

On the other hand, managing emotions may also be related to malevolent creative performance. Dark creativity involves the process of malevolent creative ideation (Cropley *et al.*, 2008; Cropley, 2010). Individuals with high MC may have the tendency to be activated with proactive emotions (i.e. anger) in response to other people’s doing or in anticipation of a psychological reward from other people’s suffering. Anger can explain the unique, non-overlapping variance in the capacity to implement MC (Perchtold-Stefan *et al.*, 2021). Similarly, Cheng *et al.* (2021a) investigated the effects of anger on MC. The results showed that idea fluency and originality were higher in the group experiencing anger than those in the group experiencing neutral emotion. Cheng *et al.* (2021b) examined whether emotional regulation could modulate such an effect. They determined that cognitive reappraisal and expression inhibition can lower anger and decrease malevolent creative performance. Accordingly, managing negative emotions may be a useful way of inhibiting MC.

The neural correlates of emotion identification are related to the postcentral gyrus (PCG) (Bufalari *et al.*, 2007; Bertsch *et al.*, 2013; Kropf *et al.*, 2019). The PCG is known to perceive primary sensations and house the secondary somatosensory cortex, which appears to integrate somatosensory stimuli and memory formation (Chen *et al.*, 2008). The PCG forms part of the somatosensory cortex, which is involved in emotional reactions due to empathetic responses (Bufalari *et al.*, 2007). Increased activity in the PCG appears to increase the identification of others’ feelings (Bufalari *et al.*, 2007; Kropf *et al.*, 2019). Recognition of others’ emotions also requires sensory involvement (Pitkänen, 2018; Yavuz *et al.*, 2018). In other words, the PCG is involved in the process of recognizing others’ emotions by integrating stimuli and sensations as the secondary somatosensory cortex. As mentioned above, recognizing others’ emotions is important for MC. In addition, the right hemisphere, especially the PCG, is particularly specialized in processing moral emotions (Borod *et al.*, 1998; Canli, 1999; Yoshino *et al.*, 2017), and these inductions affect malevolent creative performance (Fu and Zhang, 2022; Kapoor and Kaufman, 2022). Previous studies have also suggested that individuals with higher levels of psychopathy have a smaller volume of the PCG (Tiihonen *et al.*, 2008; Martina Ly *et al.*, 2012; Bertsch *et al.*, 2013) and higher aggression (Jonason *et al.*, 2014), which is associated with higher levels of MC (Lee and Dow, 2011). Based on the aforementioned research, it can be inferred that the right PCG is closely related to MC.

Managing emotions is closely correlated with activity in the middle frontal gyrus (MFG) (Ochsner *et al.*, 2004; Ohira *et al.*, 2006; Blair *et al.*, 2007). Previous findings have indicated the importance of the lateral frontal regions in emotional regulation (Ochsner *et al.*, 2004; Blair *et al.*, 2007). Emotional regulation is usually related to the activation of the MFG through suppression (Ohira *et al.*, 2006; Blair *et al.*, 2007). Brain imaging findings have indicated a positive connection between the MFG and the lateral superior pre-frontal gyrus, previously thought to be implicated in emotional management/regulation (Beauregard *et al.*, 2001; Ohira *et al.*, 2006). In particular, the right MFG is involved in downregulation and suppression effects, which are key processes for successful emotional regulation (Grecucci *et al.*, 2012; Engen and Anderson, 2018). As mentioned above, emotional regulation can affect malevolent creative performance. In so doing, the activation of the right MFG may also affect malevolent creative performance.

In the present study, we designed two studies to explore the following questions: does altering the activity of the right PCG and right MFG affect one’s emotional recognition and regulation function and does this in turn affect malevolent creative ideation? In Study 1, we assumed that when the activity of the right PCG is inhibited, individuals’ malevolent creative performance would be strengthened. If activity in the right PCG is not inhibited, an individual’s malevolent creative performance would weaken. In Study 2, we assumed that when the activity of the right MFG is higher, then the individual’s malevolent creative performance would be decreased. In contrast, an individual’s malevolent creative performance is enhanced when activity in the right MFG is decreased. Here, we altered the activity of the right PCG or right MFG using transcranial direct current stimulation (tDCS). The tDCS technique, a non-invasive brain stimulation method, can modulate cortical excitability. It can deliver a low-intense (0.5–2.0 mA) direct current to specific cortical areas and facilitate (anode) or inhibit (cathode) spontaneous neuronal activity (Nitsche and Paulus, 2000; Brunoni *et al.*, 2012). Research has shown that tDCS can affect creative performance by modulating the excitability of the cortical regions associated with creative thinking (Chi and Snyder, 2012; Mayseless and Shamay-Tsoory, 2015; Weinberger *et al.*, 2017; Hertenstein *et al.*, 2019; Ivancovsky *et al.*, 2019; Kleinmintz *et al.*, 2019). Ivancovsky *et al.* (2019) investigated the effect of tDCS of the inferior frontal gyrus (IFG) on creative divergent thinking. The results showed that individuals exhibited increased creative performance after receiving cathodal stimulation over the left IFG, whereas individuals exhibited poor creative performance after receiving anodal stimulation over the left IFG. Mayseless and Shamay-Tsoory (2015) found that anodal tDCS over the right IFG, coupled with cathodal tDCS over the left IFG, promoted verbal creative divergent thinking, whereas reverse stimulation did not affect verbal creative divergent thinking. Chi and Snyder (2011) investigated the effect of tDCS on the anterior temporal lobe (ATL) using the matchstick arithmetic task (a creative task) (Öllinger *et al.*, 2008). They found that anodal tDCS over the right ATL, coupled with cathodal tDCS over the left ATL, increased creative performance. In light of this, it was determined that tDCS modulates neuronal activity in brain regions correlated with creativity, which in turn affects creative performance (Weinberger *et al.*, 2017; Chrysikou *et al.*, 2021; Lifshitz-Ben-Basat and Mashal, 2021).

## Study 1

We examined whether altering the activity of the right PCG affects emotional recognition and malevolent creative performance using tDCS.

### Method

#### Participants

**Fig. 1.**
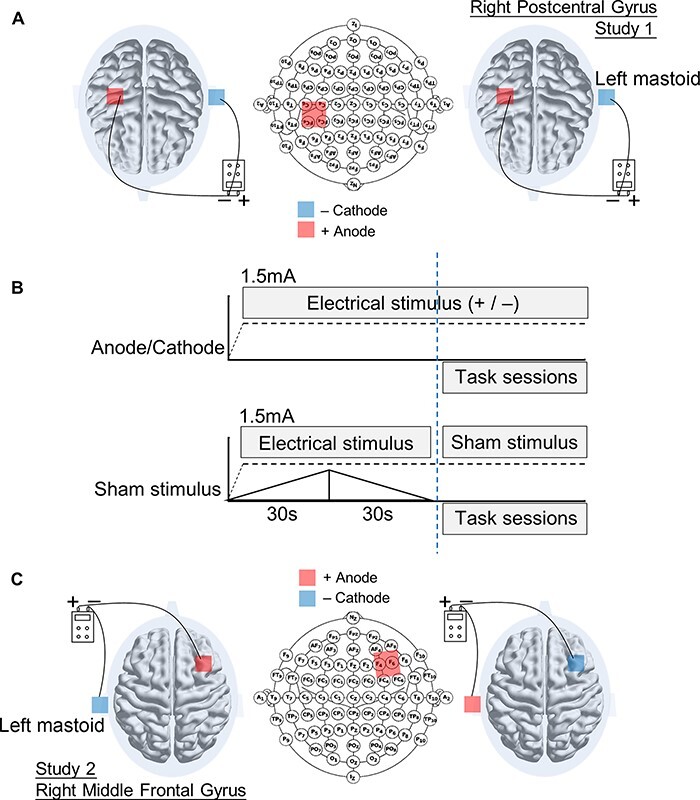
(A) Participants performed malevolent and benevolent creative tasks while being administered with tDCS over their right PCG and left mastoid. (B) The relative stimulation phase between brain regions was manipulated to be 1.5 Hz. A sham stimulation condition is applied only for the initial 30 s ramp-up and 30 s ramp-down. (C) Participants performed malevolent and benevolent creative tasks while being administered with tDCS over their right MFG and left mastoid.

One hundred and one right-handed college students were randomly assigned to three groups (anodal, cathodal and sham tDCS groups). Participants were recruited using online posters. Data of 98 participants who completed all the tasks (80 females, age = 21.11 ± 2.00 years) were entered into subsequent analysis. The anodal tDCS group (33 participants, 25 females, age = 21.31 ± 2.06 years), cathodal tDCS group (35 participants, 29 females, age = 20.97 ± 1.92 years) and sham tDCS group (30 participants, 26 females, age = 21.03 ± 2.13 years) received positive, negative and sham stimulation over the right PCG, respectively (Figure 1A). All participants had no history of alcohol or drug abuse, neurological or psychiatric abnormalities, claustrophobia or head injury. All procedures were approved by the University Committee on Human Research Protection (UCHRP) of East China Normal University (ECNU), and all participants provided informed written consent for this study.

#### Transcranial direct current stimulation

Direct current was delivered through a 1 × 1 tDCS low-intensity stimulator using a pair of saline-soaked electrodes (3 × 3 cm^2^). In the stimulation groups, participants continually received a constant current of 1.5 mA while solving creative tasks for 20 min. In the sham stimulation group, only a fade-in current (30 s) and a fade-out current (30 s) were successively delivered to the participants before the task sessions (Figure 1B). The relevant tDCS electrode was placed over the right PCG, and the non-relevant tDCS electrode was placed over the left mastoid (Figure 1A).

#### Experimental protocol

**Fig. 2.**
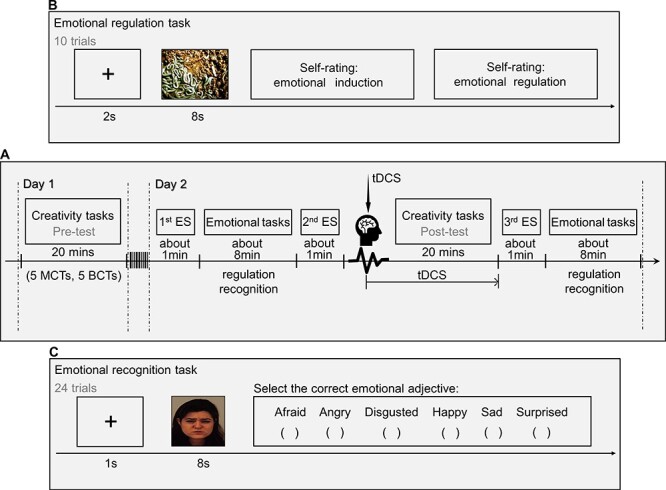
(A) The flow diagram of the data acquisition procedure. (B) The flow diagram of the emotional regulation task. (C) The flow diagram of the emotional recognition task.

All participants completed all tests within 2 days. The experimental procedure comprised two parts which were, respectively, completed in two consecutive days (Figure 2A). On Day 1 (Part 1), the participants were asked to complete a pre-test creativity task session, which comprised five 2-min malevolent creative ideation tasks (MCTs) and five 2-min benevolent creative ideation tasks (BCTs) (pre-test of creative tasks). The MCTs and BCTs were adapted from the realistic presented problems task, which asked participants to solve open-ended realistic problems originally (Okuda *et al.*, 1991; Agnoli *et al.*, 2016). For the MCTs, an example is ‘your friend, who hates to dress the same clothes as others, found that a classmate has the same coat as his. Please think of a novel manner to destroy his classmate’s coat secretly’. For the BCTs, an example is ‘your friend is too shy to meet the girl he loves. Please produce a novel manner to help him to get to know the girl naturally.’ These five MCTs or BCTs were randomly presented. Participants were required to generate as many novel responses as possible and type them into the computer.

On Day 2 (Part 2), participants completed three emotional self-reports, two emotional recognition tasks and two emotional regulation tasks. Here, the emotional tasks were used to examine whether emotional recognition/regulation was affected by tDCS stimulation. One single trial of the emotional regulation task included a jittered fixation cross (2 s), a negative image (8 s) that could induce negative emotions and a two-item self-report. Participants were required to regulate their negative emotions induced by these images. One of the self-report items was rating negative emotions induced by negative images using a 1–5 scale (1 = not at all; 5 = fully induced). The other was rating the levels of self-regulation using a 1–7 scale (1 = not at all; 7 = fully regulated). The emotional regulation task contained 10 pictures (i.e. 10 trials) from the International Affective Picture System (Bradley and Lang, 2007) (Figure 2B). The emotional recognition task presented face pictures with different emotions from the Karolinska Directed Emotional Faces (KDEF) to participants, including afraid, angry, disgusted, happy, sad and surprised faces (Goeleven *et al.*, 2008). Four sets of face pictures (i.e. four different faces) were selected. Each set consisted of six pictures referring to the above-mentioned six types of emotions (i.e. a total of 4 × 6 = 24 pictures). The presenting sequence was random among participants. One single trial of the emotional recognition task comprised a jittered fixation cross (1 s), an emotional face picture (8 s) and a choice question. Participants were asked to choose the correct emotion corresponding to the face pictures (Figure 2C). The respond time and accuracy were recorded for subsequent analysis.

First, participants were asked to complete the first emotional self-report using a 9-point Likert scale (1 = not at all; 9 = fully; ∼1 min). The items included worry, disappointment, anger, anxiety, happiness, satisfaction, stress, discouragement, relaxation, pleasure and activation level. Second, participants were required to complete the two emotional tasks (pre-test of the two emotional tasks), and these tasks were randomly presented to participants (∼8 min). Third, participants completed the second emotional self-report (∼1 min). Then, participants received tDCS and completed the post-test creativity task during electrical stimulation (20 min). Fourth, participants completed the third emotional self-report (∼1 min). Finally, participants completed the two emotional tasks again (post-test; ∼8 min). See details in Figure 2A.

#### Creative performance scoring

The performance of creativity tasks was assessed using idea fluency, originality and benevolence/malevolence (Gao *et al.*, 2022; Qiao *et al.*, 2022). Fluency was calculated based on the number of ideas. We assessed the originality, malevolence and benevolence scores by referring to the originality, malevolence and benevolence of each answer to evaluate task performance for each participant. Four trained raters independently assessed the originality, malevolence and benevolence scores for each answer to provide an index of creative, malevolent and benevolent quality using a 1–5 scale (1 = not creative/malevolent/benevolent at all; 5 = very creative/malevolent/benevolent) (Amabile, 1982). The final originality, malevolence and benevolence scores for each participant were obtained by averaging the individual ratings from all items. Individual ratings for each participant from these four raters were averaged into a single originality, malevolence or benevolence score. The Internal Consistency Coefficients (ICCs) of the four trained raters were acceptable (Supplementary Table S1).

### Results

#### Emotional self-report

One-way repeated measures Analysis of Variances (ANOVAs) using TEST (first test *vs* second test *vs* third test) as the within-subject factor were performed on worry, disappointment, anger, anxiety, happiness, satisfaction, stress, discouragement, relaxation, pleasure and activation level scores. The details of the results are presented in Table 1.

**Table 1.** Descriptive statistics and difference

	*M *± s.d.					
Emotion	First	Second	Third	*F*	*P*	$\eta _p^2$
Worry	2.64 ± 1.77	4.47 ± 2.09	3.09 ± 2.01	0.39	0.68	0.004
Disappointment	2.13 ± 1.68	2.19 ± 1.52	2.54 ± 1.87	0.19	0.83	0.002
Anger	1.46 ± 1.00	2.04 ± 1.49	2.31 ± 1.83	0.01	0.99	<0.001
Anxiety	3.38 ± 2.01	3.74 ± 1.97	3.16 ± 1.92	0.26	0.77	0.003
Happiness	4.68 ± 1.62	3.98 ± 1.62	3.86 ± 1.76	3.44	0.03*	0.035
Satisfaction	4.68 ± 1.69	3.55 ± 1.74	3.51 ± 1.82	2.66	0.07	0.027
Stress	2.97 ± 1.83	4.37 ± 2.19	2.80 ± 1.85	3.37	0.04*	0.034
Discouragement	2.40 ± 1.67	2.32 ± 1.62	2.54 ± 1.86	0.37	0.69	0.004
Relaxation	5.87 ± 1.45	4.46 ± 2.04	5.02 ± 1.99	4.42	0.01*	0.044
Pleasure	5.73 ± 1.28	4.74 ± 1.37	4.91 ± 1.64	0.78	0.46	0.008
Activation level	5.41 ± 1.41	5.78 ± 1.39	5.52 ± 1.55	1.49	0.23	0.015

*Note:* Age and gender were considered as covariates. *P < 0.05.

#### Emotional task performance

**Fig. 3.**
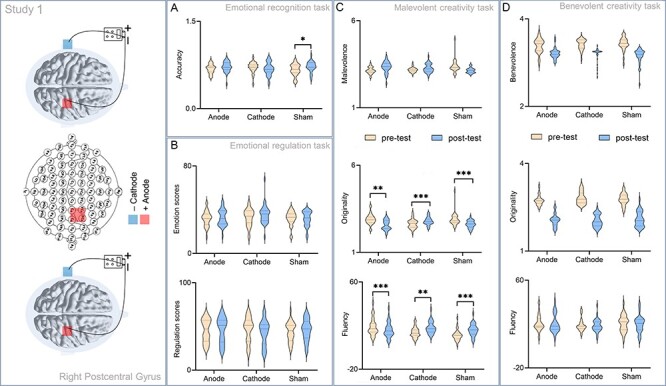
A significant difference between the pre- and post-test for emotional recognition, emotional regulation, malevolent creative and benevolent creative tasks in Study 1. tDCS was applied over the right PCG and left mastoid. (A) A significant difference between the pre- and post-test for emotional recognition tasks. (B) A significant difference between the pre- and post-test for emotional regulation tasks. (C) A significant difference between the pre- and post-test for malevolent creative tasks. (D) A significant difference between the pre- and post-test for benevolent creative tasks. ^**^P < 0.01; ^***^P < 0.0001.

Regarding the emotional recognition tasks, the response time and accuracy were submitted to two-way mix-design ANOVAs with STIMULATION (anodal, cathodal and sham stimulation) as the between-subject factor and TEST (pre-test *vs* post-test) as the within-subject factor. Results suggested no significant main effects of STIMULATION [*F*_(2,90)_ = 0.99, *P* = 0.38] and TEST [*F*_(1,90)_ = 0.57, *P* = 0.45] on the response time. Also, there was no interaction effect of TEST × STIMULATION [*F*_(2,90)_ = 0.31, *P* = 0.73] on the response time. No significant main effects of STIMULATION [*F*_(2,90)_ = 0.05, *P* = 0.96] and TEST [*F*_(1,90)_ = 0.02, *P* = 0.90] were observed on the accuracy. However, there was a significant interaction effect of TEST × STIMULATION [*F*_(2,90)_ = 4.52, *P* = 0.01, $\eta _p^2$ = 0.09] on the accuracy. Simple effect analysis revealed that the accuracy was significantly lower in the pre-test (*M* = 0.66, s.d. = 0.10) than that in the post-test (*M* = 0.72, s.d. = 0.10, *P* = 0.02) under the sham stimulation condition, while there was no difference of the accuracy between the pre-test (*M* = 0.68, s.d. = 0.08; *M* = 0.71, s.d. = 0.09) and post-test (*M* = 0.72, s.d. = 0.09, *P* = 0.08; *M* = 0.68, s.d. = 0.11, *P* = 0.13) under the anodal and cathodal stimulation conditions (Figure 3A).

Regarding the emotional regulation tasks, two-way mix-design ANOVAs using STIMULATION as the between-subject factor and TEST as the within-subject factor were performed on the emotional scores and self-regulation scores. No significant main effects of STIMULATION [*F*_(2,90)_ = 1.22, *P* = 0.30] and TEST [*F*_(1,90)_ = 0.28, *P* = 0.60] or interaction effect of TEST × STIMULATION [*F*_(2,90)_ = 1.26, *P* = 0.29] were observed on the emotional scores. There were no significant main effects of STIMULATION [*F*_(2,90)_ = 0.11, *P* = 0.89, $\eta _p^2$ = 0.003] and TEST [*F*_(1,90)_ = 0.50, *P* = 0.48] or interaction effect of TEST × STIMULATION [*F*_(2,90)_ = 2.23, *P* = 0.11] on the self-regulation scores (Figure 3B).

#### Malevolent creative task performance

The fluency, originality and malevolence scores were submitted to two-way mix-design ANOVAs with STIMULATION as the between-subject factor and TEST as the within-subject factor.

Regarding MCT fluency, no significant main effect of TEST [*F*_(1,90)_ = 1.83, *P* = 0.18] was observed on the fluency scores. We found a significant main effect of STIMULATION [*F*_(2,90)_ = 3.38, *P* = 0.04, $\eta _p^2$ = 0.003] and an interaction effect of TEST× STIMULATION [*F*_(2,90)_ = 39.44, *P* < 0.001, $\eta _p^2$  = 0.47] on the fluency scores. Simple effect analysis suggested that the fluency scores were higher in the pre-test (*M* = 18.09, s.d. = 5.52) than those in the post-test (*M* = 13.63, s.d. = 4.75, *P* < 0.001) under the anodal stimulation condition; the fluency scores were lower in the pre-test (*M* = 15.91, s.d. = 6.99; *M* = 11.13, s.d. = 5.04) than those in the post-test (*M* = 18.70, s.d. = 7.77, *P* = 0.002; *M* = 15.97, s.d. = 5.61, *P* < 0.001) under the cathodal and sham stimulation conditions (Figure 3C).

Regarding MCT originality, results showed no significant main effects of STIMULATION [*F*_(2,90)_ = 0.16, *P* = 0.85] and TEST [*F*_(1,90)_ = 0.43, *P* = 0.52, $\eta _p^2$ = 0.01] on the originality scores. We found a significant interactive effect of TEST × STIMULATION [*F*_(2,90)_ = 36.82, *P* < 0.001, $\eta _p^2$ = 0.45] on the originality scores. Simple effect analysis showed higher originality scores in the pre-test (*M* = 2.79, s.d. = 0.22) than those in the post-test (*M* = 2.64, s.d. = 0.26, *P* = 0.002) under the anodal stimulation condition, lower originality scores in the pre-test (*M* = 2.57, s.d. = 0.28) than those in the post-test (*M* = 2.89, s.d. = 0.30, *P* < 0.001) under the cathodal stimulation condition and higher originality scores in the pre-test (*M* = 2.89, s.d. = 0.37) than those in the post-test (*M* = 2.61, s.d. = 0.21, *P* < 0.001) under the sham stimulation condition (Figure 3C).

Regarding MCT malevolence, no significant main effects of STIMULATION [*F*_(2,90)_ = 0.38, *P* = 0.69] and TEST [*F*_(1,90)_ = 2.70, *P* = 0.10, $\eta _p^2$ = 0.03] or interaction effect of TEST × STIMULATION [*F*_(2,90)_ = 1.75, *P* = 0.18, $\eta _p^2$ = 0.04] were observed on the malevolence scores (Figure 3C).

#### Benevolent creative task performance

The fluency, originality and benevolence scores were submitted to two-way mix-design ANOVAs with STIMULATION as the between-subject factor and TEST as the within-subject factor.

We did not find significant main effects on the fluency [STIMULATION: *F*_(2,90)_ = 0.54, *P* = 0.58; TEST: *F*_(1,90)_ = 2.75, *P* = 0.07], originality [STIMULATION: *F*_(2,90)_ = 0.20, *P* = 0.82; TEST: *F*_(1,90)_ = 1.30, *P* = 0.26] and benevolence [STIMULATION: *F*_(2,90)_ = 0.13, *P* = 0.88; TEST: *F*_(1,90)_ = 0.16, *P* = 0.69] scores. There were no significant interactive effects of TEST × STIMULATION on the fluency [*F*_(2,90)_ = 0.02, *P* = 0.98], originality [*F*_(2,90)_ = 2.23, *P* = 0.11] and benevolence [*F*_(2,90)_ = 2.75, *P* = 0.07] scores (Figure 3D).

### Interim discussion

Study 1 aimed to explore whether activity in the right PCG affected the identification of emotions and then affected malevolent creative performance. Results showed significantly lower accuracy in the pre-test than that in the post-test under the sham stimulation condition. The MCT results showed lower fluency scores in the post-test but higher originality scores in the pre-test under the sham stimulation condition. Consistent with previous findings, the results may indicate that individuals may need more time to generate more novel ideas (Wang *et al.*, 2017, 2019). In MCTs, the originality scores were significantly higher in the pre-test than those in the post-test under the anodal and sham stimulation conditions, whereas lower originality scores were found in the pre-test under the cathodal stimulation condition. Furthermore, MCT results indicated lower fluency scores in the pre-test under the cathodal and sham stimulation conditions, whereas higher fluency scores in the pre-test under the anodal stimulation condition. That is, augmenting the right PCG reduced malevolent creative performance, whereas inhibiting it enhanced individual malevolent creative performance. Meanwhile, electrical stimulation also affected individual emotional recognition ability. In addition, we did not find a significant difference between the pre- and post-test in BCTs. However, another possibility could be that social pressure (feelings of guilt and shame) might activate the PCG during MC, which eventually impaired MC originality. It would be helpful to examine the effect of social pressure on MC performance in further research.

## Study 2

In Study 2, we examined whether altering the activity of the right MFG affects emotional regulation and malevolent creative performance using tDCS.

### Method

#### Participants

One hundred right-handed college students were randomly assigned to three groups (anodal, cathodal and sham stimulation groups). Participants were recruited using online posters. Data of 91 participants who completed all the tasks (74 females, age = 21.22 ± 2.28 years) were entered into subsequent analysis. The anodal tDCS group (32 participants, 25 females, age = 21.44 ± 2.14 years), cathodal tDCS group (29 participants, 24 females, age = 21.17 ± 2.74 years) and sham tDCS group (30 participants, 26 females, age = 21.00 ± 2.10 years) received positive, negative and sham stimulation over the right MFG, respectively (Figure 1C). All participants had no history of alcohol or drug abuse, neurological or psychiatric abnormalities, claustrophobia or head injury. All procedures were approved by the UCHRP of ECNU, and all participants provided informed written consent for this study.

#### Transcranial direct current stimulation

The tDCS procedure was similar to Study 1. The relevant tDCS electrode was placed over the right MFG, and the non-relevant tDCS electrode was placed over the left mastoid (Figure 1C).

#### Experimental protocol

The experimental protocol was similar to Study 1.

#### Scoring and statistical analysis

Scoring and statistical analysis were similar to Study 1. The ICCs are presented in Supplementary Table S2.

### Results

#### Emotional self-report

One-way repeated measures ANOVAs using TEST (first test *vs* second test *vs* third test) as the within-subject factor were performed on worry, disappointment, anger, anxiety, happiness, satisfaction, stress, discouragement, relaxation, pleasure and activation level scores. Results showed no significant main effect of TEST on worry [*F*_(2,176)_ = 0.46, *P* = 0.63], disappointment [*F*_(2,176)_ = 0.48, *P* = 0.59], anger [*F*_(2,176)_ = 1.21, *P* = 0.30], calmness [*F*_(2,176)_ = 1.21, *P* = 0.30], anxiety [*F*_(2,176)_ = 0.37, *P* = 0.70], happiness [*F*_(2,176)_ = 0.95, *P* = 0.39], satisfaction [*F*_(2,176)_ = 0.07, *P* = 0.93], stress [*F*_(2,176)_ = 0.83, *P* = 0.43], discouragement [*F*_(2,176)_ = 0.28, *P* = 0.76], relaxation [*F*_(2,176)_ = 0.48, *P* = 0.62], pleasure [*F*_(2,176)_ = 0.55, *P* = 0.55] and activation level [*F*_(2,176)_ = 0.27, *P* = 0.77].

#### Emotional task performance

**Fig. 4.**
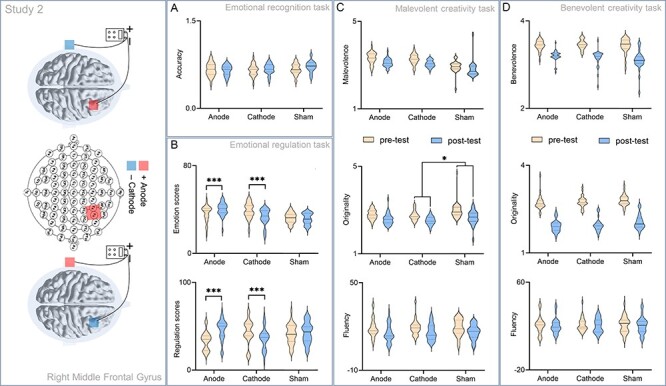
A significant difference between the pre- and post-test for emotional recognition, emotional regulation, malevolent creative and benevolent creative tasks in Study 2. tDCS was applied over their right MFG and left mastoid. (A) A significant difference between the pre- and post-test for emotional recognition tasks. (B) A significant difference between the pre- and post-test for emotional regulation tasks. (C) A significant difference between the pre- and post-test for malevolent creative tasks. (D) A significant difference between the pre- and post-test for benevolent creative tasks. *P < 0.05; ^***^P < 0.0001.

Regarding emotional recognition tasks, we performed two-way mix-design ANOVAs using STIMULATION (anodal, cathodal and sham stimulations) as the between-subject factor and TEST (pre-test *vs* post-test) as the within-subject factor on the response time and accuracy. No significant effects were observed (Figure 4A).

Regarding emotional regulation tasks, the emotional scores and self-regulation scores were submitted to two-way mix-design ANOVAs with STIMULATION as the between-subject factor and TEST as the within-subject factor. Results suggested no significant main effect of TEST [*F*_(1,86)_ = 0.42, *P* = 0.52] on the emotional scores. We found a significant main effect of STIMULATION [*F*_(2,86)_ = 8.63, *P* < 0.001, $\eta _p^2$ =  0.17] and an interaction effect of TEST × STIMULATION [*F*_(2,86)_ = 26.64, *P* < 0.001, $\eta _p^2$ = 0.38] on the emotional scores. Simple effect analysis showed lower emotional scores in the pre-test (*M* = 36.43, s.d. = 7.34) than those in the post-test (*M* = 40.22, s.d. = 5.91, *P* < 0.001) under the anodal stimulation condition; the emotional scores were higher in the pre-test (*M* = 38.97, s.d.  = 6.76) than those in the post-test (*M* = 33.14, s.d.  = 7.36, *P* < 0.001) under the cathodal stimulation condition. There was no significant difference between the pre-test (*M* = 36.06, s.d.  = 6.79) and post-test (*M* = 35.47, s.d.  = 7.11, *P* = 0.76) on the emotional scores under the sham stimulation condition (Figure 4B).

No significant main effects of STIMULATION [*F*_(2,86)_ = 0.46, *P* = 0.63] and TEST [*F*_(1,86)_ = 2.16, *P* = 0.15] were found on the self-regulation scores. We found a significant interaction effect of TEST × STIMULATION on the self-regulation scores [*F*_(2,86)_ = 31.34, *P* < 0.001, $\eta _p^2$ = 0.42]. Simple effect analysis showed lower self-regulation scores in the pre-test (*M* = 33.41, s.d. = 10.54) than those in the post-test (*M* = 45.69, s.d. = 12.09, *P* < 0.001) under the anodal stimulation condition; the self-regulation scores were higher in the pre-test (*M* = 43.24, s.d. = 13.75) than those in the post-test (*M* = 35.72, s.d. = 11.89, *P* < 0.001) under the cathodal stimulation condition. There was no significant difference between the pre-test (*M* = 41.07, s.d. = 11.27) and post-test (*M* = 42.47, s.d. = 11.86, *P* = 0.49) under the sham stimulation condition on the self-regulation scores (Figure 4B).

#### Malevolent creative task performance

We performed two-way mix-design ANOVAs using STIMULATION as the between-subject factor and TEST as the within-subject factor on the fluency, originality and malevolence scores. Results showed no significant main effects of STIMULATION [*F*_(2,86)_ = 0.12, *P* = 0.89] and TEST [*F*_(1,86)_ = 0.66, *P* = 0.42] on the fluency scores. Results showed a significant main effect of STIMULATION [*F*_(2,86)_ = 5.42, *P* = 0.01, $\eta _p^2$ = 0.11] and no significant main effect of TEST [*F*_(1,86)_ = 0.68, *P* = 0.41] on the originality scores. Simple effect analysis showed lower originality scores under the cathodal simulation condition (*M* = 2.63, s.d. = 0.24) than those under the sham simulation condition (*M* = 2.82, *SD* = 0.43). We found no significant difference in the other conditions. No significant main effects of STIMULATION [*F*_(2,86)_ = 2.28, *P* = 0.11] and TEST [*F*_(1,86)_ = 1.88, *P* = 0.17] were found on the malevolence scores. We found no significant interaction effects of TEST × STIMULATION on the fluency [*F*_(2,86)_ = 0.06, *P* = 0.95], originality [*F*_(2,86)_ = 1.63, *P* = 0.20] and malevolence [*F*_(2,86)_ = 0.24, *P* = 0.79] scores (Figure 4C).

#### Benevolent creative task performance

The fluency, originality and benevolence scores were submitted to two-way mix-design ANOVAs with STIMULATION as the between-subject factor and TEST as the within-subject factor. Results showed no significant main effects of SIMULATION on the fluency [*F*_(2,86)_ = 0.08, *P* = 0.92], originality [*F*_(2,86)_ = 2.57, *P* = 0.08] and benevolence [*F*_(2,86)_ = 0.84, *P* = 0.43] scores. We found a significant main effect of TEST on the fluency [*F*_(1,86)_ = 4.70, *P* = 0.03, $\eta _p^2$ = 0.05] and originality [*F*_(1,86)_ = 12.72, *P* = 0.001, $\eta _p^2$ = 0.13] scores. Simple effect analysis showed higher fluency and originality scores in the pre-test (*M* = 20.87, s.d. = 7.21; *M* = 2.75, s.d. = 0.20) than those in the post-test (*M* = 20.29, s.d. = 6.93; *M* = 1.98, s.d. = 0.23). No significant main effect was found on the benevolence scores [*F*_(1,86)_ = 0.41, *P* = 0.52]. Results showed no significant interaction effects of TEST × STIMULATION on the fluency [*F*_(2,86)_ = 0.03, *P* = 0.97], originality [*F*_(2,86)_ = 1.20, *P* = 0.31] and benevolence [*F*_(2,86)_ = 1.98, *P* = 0.14] scores (Figure 4D).

### Interim discussion

In Study 2, we aimed to explore whether inhibiting activity in the right MFG may decline emotional regulation but enhanced individuals’ malevolent creative performance, whereas enhancing it may lead to a decline in malevolent creative performance.

We found a significant difference between the pre- and post-test on emotional regulation. However, in the creative tasks, results showed no significant interaction effect of TEST × STIMULATION on the fluency, originality and malevolence/benevolence scores. Our results were inconsistent with our hypothesis. Although inhibiting activity in the right MFG may decline emotional regulation, individuals’ creative performance was not affected. We assumed that the right MFG was involved in the process of emotional regulation. Nevertheless, the right MFG is involved in many other high-level cognitive functions such as working memory (Mottaghy *et al.*, 2003; Jolles *et al.*, 2013; Subramaniam *et al.*, 2014) and attention (Vossel *et al.*, 2014; Agmon *et al.*, 2021), which are closely associated with creativity (Vartanian, 2009; Benedek *et al.*, 2014; Zabelina, 2018). From this point of view, it seemed that if activity in the MFG was enhanced, creative performance should also be enhanced, as this result was contrary to our hypothesis. We speculated that augmenting activity in the MFG enhanced individuals’ emotional regulation but reduced malevolent creative performance. Under the circumstances, individuals’ malevolent creative performance was not changed because the right MFG was involved in both emotional regulation and creative cognition processing.

## Discussion

This study explored the relationship between malevolent creative performance and the activity of the right PCG or right MFG using the tDCS technique. Study 1 showed that augmenting the right PCG using tDCS decreased the performance of MC, whereas inhibiting it enhanced the performance of MC. Study 2 showed that stimulating the right MFG with tDCS did not affect an individual’s performance of MC.

Study 1 showed that augmenting the activity of the right PCG impaired individual malevolent creative performance, whereas inhibiting this brain region enhanced malevolent creative performance. Previous research has indicated that the right PCG is involved in the identification of others’ emotional expressions (Bufalari *et al.*, 2007; Morawetz *et al.*, 2020). The primary and secondary somatosensory cortices have a closed relation in facial emotional recognition, helping individuals construct somatosensory images related to emotions (Adolphs *et al.*, 2000; Adolphs, 2002). As one of the primary and secondary somatosensory cortices, the PCG is also involved in emotional recognition (Satoh *et al.*, 2002; Bufalari *et al.*, 2007; Chen *et al.*, 2008). Study 1 showed significantly lower accuracy in the emotional recognition task in the pre-test than in the post-test under the sham stimulation condition, possibly owing to the practice effect. However, intriguingly, stimulating the right PCG seemed to counteract the supposed practice effect in the other two conditions. In other words, the practice effect vanished under both the anodal and cathodal stimulation conditions, and even lower accuracy was observed in the post-test than in the pre-test under the cathodal stimulation condition. We did not find a significant difference between the pre- and post-test in emotional recognition tasks. One possible reason may be due to the face pictures that we chose for the tasks. The face pictures that we chose may need to be more suitable for the tasks because the face pictures were randomly selected from the KDEF, and we did not check the accuracy of emotional recognition in Chinese. Choosing more suitable stimulus materials is a possible solution for future exploration.

The AMORAL model proposes that socio-emotional skills, such as emotional recognition, are important contributing factors to MC (Kapoor and Kaufman, 2022). Moral emotions (i.e. sympathy and guilt) can promote prosocial behaviors (Ferguson and Branscombe, 2010; Rees *et al.*, 2015; Hurst and Sintov, 2022). Sympathy, defined as understanding and sharing of another individual’s feelings, is associated with emotional recognition. Sympathy leads individuals to react sympathetically to others’ suffering, and individuals with higher sympathy tend to find novel and positive solutions to promote prosocial behavior (Rees *et al.*, 2015; Yang and Yang, 2016). Sympathy may interfere with malevolent creative performance (Eisenberg, 2000; Malti *et al.*, 2013). Similarly, recognizing others’ emotional expressions or sensations, which is linked to the activity of the right PCG (Adolphs *et al.*, 2000; Bufalari *et al.*, 2007), may cause specific negative moral emotions (i.e. guilt and shame) and thus prevent malevolent actions. Individuals are less likely to create malevolent consequences when experiencing guilt and shame (Rees *et al.*, 2015; Kapoor and Kaufman, 2022). Consistent with these findings, Perchtold-Stefan *et al.* (2022) found that higher levels of MC corresponded with relative increases in electroencephalogram coherence during others’ expressions of anger. Individuals recognized others’ anger with greater emotional detachment, possibly indicating that they were unperturbed by the potential consequences of their malevolent actions (Perchtold-Stefan *et al.*, 2022). This combination suggests that malevolent creative performance may be affected by the identification of others’ emotions. Augmenting the right PCG weakens malevolent creative performance, most likely through enhancing the identification of others’ emotions. Although no significant difference was observed in emotional recognition tasks under electrical stimulation conditions, it was possible that MC was still affected due to negative social emotions, which referred to provide immediate and salient feedback on our social and moral acceptability, such as guilt, shame and sympathy (Haidt, 2003a; Tangney *et al.*, 2007). Meanwhile, such emotions also provide the motivational force to do good and avoid doing bad (Kroll and Egan, 2004). However, this study did not measure these social emotions. We should examine emotional recognition ability directly and corresponding changes of social emotions in subsequent research.

Although the results showed that malevolent creative performance decreased significantly when the activity of the right PCG was enhanced, there was no significant difference in the pre-test and post-test of benevolent creative performance. This may indicate that the activity of the right PCG, which is closely linked to emotional recognition (Bufalari *et al.*, 2007), had a greater influence on MC than on other forms of creativity. However, this influence needs to be further examined in future studies.

The AMORAL model also proposed that emotional regulation is closely interlinked with MC (Kapoor and Kaufman, 2022). Study 2 explored whether inhibiting activity in the right MFG reduced emotional regulation and, as a consequence, increased malevolent creative performance. Although we found a significant difference between the pre- and post-test in the emotional regulation tasks, results of the creative tasks showed no significant difference between the pre- and post-test for fluency, originality and malevolence/benevolence. These results are inconsistent with our hypotheses. That is, although inhibiting the activity of the right MFG may reduce emotional regulation, individuals’ creative performance is not affected.

The right MFG is involved in emotional regulation. Augmenting the activity of the MFG enhances individuals’ emotion regulation ability, and individuals may experience more negative emotions in the process of malevolent creative ideation. Previous findings indicate that negative feelings affect MC (Cheng *et al.*, 2021a,b). For example, Cheng *et al.* (2021a,b) found that fluency and originality were higher in the anger group than those in the neutral group and that cognitive reappraisal and expression inhibition can decrease malevolent creative performance. Negative emotions also lead to the experience of guilt, thus promoting positive behaviors (Rees *et al.*, 2015), which are not conducive to malevolent creative ideation. Given this, managing negative emotions may reduce malevolent creative performance. Nevertheless, the right MFG is involved in many other high-level cognitive functions such as working memory (Jolles *et al.*, 2013; Subramaniam *et al.*, 2014) and attention (Vossel *et al.*, 2014; Agmon *et al.*, 2021). Such cognitive functions are closely related to creativity (Benedek *et al.*, 2014; Zabelina, 2018). Accordingly, it seems that if MFG activity was enhanced, creative performance should also be enhanced, which is contrary to the influence of emotional regulation on malevolent creative performance. Under these circumstances, an individual’s malevolent creative performance does not change because the right MFG is involved in both emotional regulation and creative cognitive processing. In addition, the right MFG is less active under certain emotional states. It is possible that the emotional tasks preceding the electrical stimulation and creativity task created a specific state-dependent brain response. In this case, the activation or deactivation of the right MFG did not affect MC performance (Bogdanov and Schwabe, 2016).

Some limitations of this study warrant further discussion. First, only college students were recruited as participants; as such, it is necessary to further examine whether these findings can be generalized to other groups. Second, given that the right MFG is also involved in a variety of high-level cognitive functions, whether stimulating the right MFG affects other cognitive functions should be further examined. Third, although we stimulated the target brain regions using tDCS, it was unclear whether the activity of the target brain regions was actually enhanced or inhibited after receiving electrical stimulation. Therefore, it would be helpful to utilize several neuroimaging techniques (e.g. functional magnetic resonance imaging and functional near-infrared spectroscopy) to assess the activity of the target brain regions after stimulation in future research.

## Conclusion

Using the tDCS technique, we found when enhancing the activity of the right PCG reduced malevolent creative performance, it was possible due to individuals’ emotional recognition improved. That is, individuals were affected by sympathy, guilt and shame; thus, their malevolent creative performance would be decreased. In a word, malevolent creative performance may be affected by the identification of others’ emotions, which was relatively associated with the activity of the right PCG.

## Supplementary Material

nsad019_Supp

## Data Availability

The data and code used to support the findings of this study are available from the corresponding author upon request. The data can only be used for research. If the associated research is to be published, the statement ‘The data and code were acquired from the Shanghai Key Laboratory of Mental Health and Psychological Crisis Intervention, School of Psychology and Cognitive Science, East China Normal University’ is required in the manuscript’.

## References

[R1] Adolphs R. (2002). Recognizing emotion from facial expressions: psychological and neurological mechanisms. *Behavioral and Cognitive Neuroscience Reviews*, 1, 21–62. 17715585 10.1177/1534582302001001003

[R2] Adolphs R. , Damasio H., Tranel D., Cooper G., Damasio A.R. (2000). A role for somatosensory cortices in the visual recognition of emotion as revealed by three-dimensional lesion mapping. *The Journal of Neuroscience*, 20, 2683–90. 10729349 10.1523/JNEUROSCI.20-07-02683.2000 PMC6772225

[R3] Agmon G. , Yahav P.H.-S., Ben-Shachar M., Golumbic E.Z. (2021). Attention to speech: mapping distributed and selective attention systems. *Cerebral Cortex*, 32, 3763–76. 10.1093/cercor/bhab446 34875678

[R4] Agnoli S. , Corazza G.E., Runco M.A. (2016). Estimating creativity with a multiple-measurement approach within scientific and artistic domains. *Creativity Research Journal*, 28, 171–6.

[R5] Amabile T.M. (1982). Social psychology of creativity: a consensual assessment technique. *Journal of Personality and Social Psychology*, 43, 997–1013.

[R6] Babcock B.A. (1983). Defending the guilty. *Cleveland State Law Review*, 32, 175–87.

[R7] Beauregard M. , Lévesque J., Bourgouin P. (2001). Neural correlates of conscious self-regulation of emotion. *The Journal of Neuroscience*, 21, RC165. 10.1523/JNEUROSCI.21-18-j0001.2001 PMC6763007 11549754

[R8] Benedek M. , Jauk E., Sommer M., Arendasy M., Neubauer A.C. (2014). Intelligence, creativity, and cognitive control: the common and differential involvement of executive functions in intelligence and creativity. *Intelligence*, 46, 73–83. 25278640 10.1016/j.intell.2014.05.007 PMC4175011

[R9] Bertsch K. , Grothe M., Prehn K., et al. (2013). Brain volumes differ between diagnostic groups of violent criminal offenders. *European Archives of Psychiatry and Clinical Neuroscience*, 263, 593–606. 23381548 10.1007/s00406-013-0391-6

[R10] Blair K.S. , Smith B.W., Mitchell D.G.V., et al. (2007). Modulation of emotion by cognition and cognition by emotion. *NeuroImage*, 35, 430–40. 17239620 10.1016/j.neuroimage.2006.11.048 PMC1862681

[R11] Bogdanov M. , Schwabe L. (2016). Transcranial stimulation of the dorsolateral prefrontal cortex prevents stress-induced working memory deficits. *The Journal of Neuroscience*, 36, 1429–37. 26818528 10.1523/JNEUROSCI.3687-15.2016 PMC6604824

[R12] Borod J.C. , Cicero B.A., Obler L.K., et al. (1998). Right hemisphere emotional perception: evidence across multiple channels. *Neuropsychology*, 12, 446–58. 9673999 10.1037//0894-4105.12.3.446

[R13] Bradley M.M. Lang P.J. (2007). The International Affective Picture System (IAPS) in the study of emotion and attention. In: Coan J.A., Allen J.B., editors. *Handbook of Emotion Elicitation and Assessment*, New York: Oxford University Press, 29–46.

[R14] Brunoni A.R. , Nitsche M.A., Bolognini N., et al. (2012). Clinical research with transcranial direct current stimulation (tDCS): challenges and future directions. *Brain Stimulation*, 5, 175–95. 22037126 10.1016/j.brs.2011.03.002 PMC3270156

[R15] Bufalari I. , Aprile T., Avenanti A., Di Russo F., Aglioti S.M. (2007). Empathy for pain and touch in the human somatosensory cortex. *Cerebral Cortex*, 17, 2553–61. 17205974 10.1093/cercor/bhl161

[R16] Canli T. (1999). Hemispheric asymmetry in the experience of emotion: a perspective from functional imaging. *The Neuroscientist*, 5, 201–7.

[R17] Carlo G. , Mestre M.V., Samper P., Tur A., Armenta B.E. (2010). The longitudinal relations among dimensions of parenting styles, sympathy, prosocial moral reasoning, and prosocial behaviors. *International Journal of Behavioral Development*, 35, 116–24.

[R18] Chen T.L. , Babiloni C., Ferretti A., et al. (2008). Human secondary somatosensory cortex is involved in the processing of somatosensory rare stimuli: an fMRI study. *NeuroImage*, 40, 1765–71. 18329293 10.1016/j.neuroimage.2008.01.020

[R19] Cheng R. , Lu K., Hao N. (2021a). The effect of anger on different forms of malevolent creative performance. *Journal of Psychological Science*, 44, 1336–45.

[R20] Cheng R. , Lu K., Hao N. (2021b). The effect of anger on malevolent creativity and strategies for its emotion regulation. *Acta Psychologica Sinica*, 53, 847–60.

[R21] Chi R.P. , Snyder A.W. (2011). Facilitate insight by non-invasive brain stimulation. *PLoS One*, 6, e16655. 10.1371/journal.pone.0016655 PMC3032738 21311746

[R22] Chi R.P. , Snyder A.W. (2012). Brain stimulation enables the solution of an inherently difficult problem. *Neuroscience Letters*, 515, 121–4. 22440856 10.1016/j.neulet.2012.03.012

[R23] Chrysikou E.G. Morrow H.M. , Flohrschutz A., Denney L. (2021). Augmenting ideational fluency in a creativity task across multiple transcranial direct current stimulation montages. *Scientific Reports*, 11, 8874. 10.1038/s41598-021-85804-3 PMC8065129 33893329

[R24] Cropley D.H. (2010). Malevolent innovation: opposing the dark side of creativity. In: Cropley, A.J., Cropley, D.H., Kaufman, J.C., Runco, M.A., editors. *The Dark Side of Creativity*, Cambridge: Cambridge University Press, 339–59.

[R25] Cropley D. , Kaufman J., Cropley A. (2008). Malevolent creativity: a functional model of creativity in terrorism and crime. *Creativity Research Journal*, 20, 105–15.

[R26] Eisenberg N. (2000). Emotion, regulation, and moral development. *Annual Review of Psychology*, 51, 665–97. 10.1146/annurev.psych.51.1.665 10751984

[R27] Eisenberg N. , Fabes R.A., Miller P.A., et al. (1989). Relation of sympathy and personal distress to prosocial behavior: a multimethod study. *Journal of Personality and Social Psychology*, 57, 55–66. 2754604 10.1037//0022-3514.57.1.55

[R28] Eisenberg N. , Miller P. (1987). The relation of empathy to prosocial and related behaviors. *Psychological Bulletin*, 101, 91–119. 3562705

[R29] Engen H.G. , Anderson M.C. (2018). Memory control: a fundamental mechanism of emotion regulation. *Trends in Cognitive Sciences*, 22, 982–95. 30122359 10.1016/j.tics.2018.07.015 PMC6198111

[R30] Ferguson M.A. , Branscombe N.R. (2010). Collective guilt mediates the effect of beliefs about global warming on willingness to engage in mitigation behavior. *Journal of Environmental Psychology*, 30, 135–42.

[R31] Fu H. , Zhang Z. (2022). The inhibitory effect of moral emotions on malevolent creativity: exploring the mediation role of emotional valence and prosocial behavior. *Frontiers in Psychology*, 13, 945848. 10.3389/fpsyg.2022.945848 PMC9430337 36059755

[R32] Gao Z. , Cheng L., Li J., Chen Q., Hao N. (2022). The dark side of creativity: neural correlates of malevolent creative idea generation. *Neuropsychologia*, 167, 108164. 10.1016/j.neuropsychologia.2022.108164 35085597

[R33] Gino F. , Ariely D. (2012). The dark side of creativity: original thinkers can be more dishonest. *Journal of Personality and Social Psychology*, 102, 445–59. 22121888 10.1037/a0026406

[R34] Goeleven E. , De Raedt R., Leyman L., Verschuere B. (2008). The Karolinska Directed Emotional Faces: a validation study. *Cognition & Emotion*, 22, 1094–118.

[R35] Grecucci A. , Giorgetta C., Van’t Wout M., Bonini N., Sanfey A.G. (2012). Reappraising the ultimatum: an fMRI study of emotion regulation and decision making. *Cerebral Cortex*, 23, 399–410. 22368088 10.1093/cercor/bhs028

[R36] Grueneisen S. , Warneken F. (2022). The development of prosocial behavior—from sympathy to strategy. *Current Opinion in Psychology*, 43, 323–8. 34530222 10.1016/j.copsyc.2021.08.005

[R37] Haidt J. (2003a). Elevation and the positive psychology of morality In: Keyes C.L.M., Haidt J., editors. *Flourishing: Positive Psychology and the Life Well-Lived*, New York: American Psychological Association, 275–89.

[R38] Haidt J. (2003b). The moral emotions. In: Davidson R.J., Scherer K.R., Goldsmith H.H., editors. *Handbook of Affective Sciences*, Washington: Oxford University Press, 852–70.

[R39] Hao N. , Qiao X., Cheng R., Lu K., Tang M., Runco M.A. (2020). Approach motivational orientation enhances malevolent creativity. *Acta Psychologica*, 203, 102985. 10.1016/j.actpsy.2019.102985 31863973

[R40] Harris D.J. , Reiter-Palmon R., Kaufman J.C. (2013). The effect of emotional intelligence and task type on malevolent creativity. *Psychology of Aesthetics, Creativity, and the Arts*, 7, 237–44.

[R41] Hertenstein E. , Waibel E., Frase L., et al. (2019). Modulation of creativity by transcranial direct current stimulation. *Brain Stimulation*, 12, 1213–21. 31231043 10.1016/j.brs.2019.06.004

[R42] Hurst K.F. , Sintov N.D. (2022). Guilt consistently motivates pro-environmental outcomes while pride depends on context. *Journal of Environmental Psychology*, 80, 101776.

[R43] Ivancovsky T. , Kurman J., Morio H., Shamay-Tsoory S. (2019). Transcranial direct current stimulation (tDCS) targeting the left inferior frontal gyrus: effects on creativity across cultures. *Social Neuroscience*, 14, 277–85. 29641936 10.1080/17470919.2018.1464505

[R44] Jolles D.D. , van Buchem M.A., Crone E.A., Rombouts S.A.R.B. (2013). Functional brain connectivity at rest changes after working memory training. *Human Brain Mapping*, 34, 396–406. 22076823 10.1002/hbm.21444 PMC6870317

[R45] Jonason P.K. , Wee S., Li N.P., Jackson C. (2014). Occupational niches and the Dark Triad traits. *Personality and Individual Differences*, 69, 119–23.

[R46] Kapoor H. , Kaufman J.C. (2022). The evil within: the AMORAL model of dark creativity. *Theory & Psychology*, 32, 467–90.

[R47] Kleinmintz O.M. , Ivancovsky T., Shamay-Tsoory S.G. (2019). The two-fold model of creativity: the neural underpinnings of the generation and evaluation of creative ideas. *Current Opinion in Behavioral Sciences*, 27, 131–8.

[R48] Kroll J. , Egan E. (2004). Psychiatry, moral worry, and the moral emotions. *Journal of Psychiatric Practice*, 10, 352–60. 15583516 10.1097/00131746-200411000-00003

[R49] Kropf E. , Syan S.K., Minuzzi L., Frey B.N. (2019). From anatomy to function: the role of the somatosensory cortex in emotional regulation. *Brazilian Journal of Psychiatry*, 41, 261–9. 30540029 10.1590/1516-4446-2018-0183 PMC6794131

[R50] Lee S.A. , Dow G.T. (2011). Malevolent creativity: does personality influence malicious divergent thinking? *Creativity Research Journal*, 23, 73–82.

[R51] Lifshitz-Ben-Basat A. , Mashal N. (2021). Enhancing creativity by altering the frontoparietal control network functioning using transcranial direct current stimulation. *Experimental Brain Research*, 239, 613–26. 33388813 10.1007/s00221-020-06023-2

[R52] Malti T. , Eisenberg N., Kim H., Buchmann M. (2013). Developmental trajectories of sympathy, moral emotion attributions, and moral reasoning: the role of parental support. *Social Development*, 22, 773–93.

[R53] Martina Ly B.S. , Motzkin J.C., Philippi C.L., et al. (2012). Cortical thinning in psychopathy. *American Journal of Psychiatry*, 169, 743–9. 22581200 10.1176/appi.ajp.2012.11111627 PMC3815681

[R54] Mayseless N. , Shamay-Tsoory S. (2015). Enhancing verbal creativity: modulating creativity by altering the balance between right and left inferior frontal gyrus with tDCS. *Neuroscience*, 291, 167–76. 25659343 10.1016/j.neuroscience.2015.01.061

[R55] Morawetz C. , Riedel M.C., Salo T., et al. (2020). Multiple large-scale neural networks underlying emotion regulation. *Neuroscience and Biobehavioral Reviews*, 116, 382–95. 32659287 10.1016/j.neubiorev.2020.07.001

[R56] Mottaghy F.M. , Pascual-Leone A., Kemna L.J., et al. (2003). Modulation of a brain–behavior relationship in verbal working memory by rTMS. *Cognitive Brain Research*, 15, 241–9. 12527098 10.1016/s0926-6410(02)00196-9

[R57] Nitsche M.A. , Paulus W. (2000). Excitability changes induced in the human motor cortex by weak transcranial direct current stimulation. *The Journal of Physiology*, 527(Pt 3), 633–9. 10990547 10.1111/j.1469-7793.2000.t01-1-00633.x PMC2270099

[R58] Ochsner K.N. , Ray R.D., Cooper J.C., et al. (2004). For better or for worse: neural systems supporting the cognitive down- and up-regulation of negative emotion. *NeuroImage*, 23, 483–99. 15488398 10.1016/j.neuroimage.2004.06.030

[R59] Ohira H. , Nomura M., Ichikawa N., et al. (2006). Association of neural and physiological responses during voluntary emotion suppression. *NeuroImage*, 29, 721–33. 16249100 10.1016/j.neuroimage.2005.08.047

[R60] Okuda S.M. , Runco M.A., Berger D.E. (1991). Creativity and the finding and solving of real-world problems. *Journal of Psychoeducational Assessment*, 9, 45–53.

[R61] Öllinger M. , Jones G., Knoblich G. (2008). Investigating the effect of mental set on insight problem solving. *Experimental Psychology*, 55, 269–82. 18683624 10.1027/1618-3169.55.4.269

[R62] Perchtold-Stefan C.M. , Fink A., Rominger C., Papousek I. (2021). Creative, antagonistic, and angry? Exploring the roots of malevolent creativity with a real-world idea generation task. *The Journal of Creative Behavior*, 55, 710–22. 34690361 10.1002/jocb.484 PMC8518065

[R63] Perchtold-Stefan C.M. , Fink A., Rominger C., Szabó E., Papousek I. (2022). Enjoying others’ distress and indifferent to threat? Changes in prefrontal-posterior coupling during social-emotional processing are linked to malevolent creativity. *Brain and Cognition*, 163, 105913. 10.1016/j.bandc.2022.105913 36087513

[R64] Pitkänen M. (2018). On emotions as sensory percepts of the state of magnetic body. *Journal of Consciousness Exploration & Research*, 9, 250–68.

[R65] Qiao X. , Lu K., Teng J., Gao Z., Hao N. (2022). Middle occipital area differentially associates with malevolent versus benevolent creativity: an fNIRS investigation. *Social Neuroscience* 17, 127–42. 35114089 10.1080/17470919.2022.2038261

[R66] Rees J.H. , Klug S., Bamberg S. (2015). Guilty conscience: motivating pro-environmental behavior by inducing negative moral emotions. *Climatic Change*, 130, 439–52.

[R67] Runco M.A. , Jaeger G.J. (2012). The standard definition of creativity. *Creativity Research Journal*, 24, 92–6.

[R68] Satoh M. , Terada S., Onouchi K., Takeda K., Kuzuhara S. (2002). Somatosensory and skin temperature disturbances caused by infarction of the postcentral gyrus. *Journal of Neurology*, 249, 1404–8. 12382157 10.1007/s00415-002-0853-7

[R69] Subramaniam K. , Luks T.L., Garrett C., et al. (2014). Intensive cognitive training in schizophrenia enhances working memory and associated prefrontal cortical efficiency in a manner that drives long-term functional gains. *NeuroImage*, 99, 281–92. 24867353 10.1016/j.neuroimage.2014.05.057 PMC4498800

[R70] Tangney J.P. , Stuewig J., Mashek D.J. (2007). Moral emotions and moral behavior. *Annual Review of Psychology*, 58, 345–72. 10.1146/annurev.psych.56.091103.070145 PMC3083636 16953797

[R71] Tiihonen J. , Rossi R., Laakso M.P., et al. (2008). Brain anatomy of persistent violent offenders: more rather than less. *Psychiatry Research: Neuroimaging*, 163, 201–12. 10.1016/j.pscychresns.2007.08.012 18662866

[R72] Vartanian O. (2009). Variable attention facilitates creative problem solving. *Psychology of Aesthetics, Creativity, and the Arts*, 3, 57–9.

[R73] Vossel S. , Geng J.J., Fink G.R. (2014). Dorsal and ventral attention systems: distinct neural circuits but collaborative roles. *The Neuroscientist*, 20, 150–9. 23835449 10.1177/1073858413494269 PMC4107817

[R74] Wang M. , Hao N., Ku Y., Grabner R.H., Fink A. (2017). Neural correlates of serial order effect in verbal divergent thinking. *Neuropsychologia*, 99, 92–100. 28259772 10.1016/j.neuropsychologia.2017.03.001

[R75] Wang X. , He Y., Lu K., Deng C., Qiao X., Hao N. (2019). How does the embodied metaphor affect creative thinking? *NeuroImage*, 202, 116114. 10.1016/j.neuroimage.2019.116114 31442486

[R76] Weinberger A.B. , Green A.E., Chrysikou E.G. (2017). Using transcranial direct current stimulation to enhance creative cognition: interactions between task, polarity, and stimulation site. *Frontiers in Human Neuroscience*, 11, 246. 10.3389/fnhum.2017.00246 PMC5432551 28559804

[R77] Yang H. , Yang S. (2016). Sympathy fuels creativity: the beneficial effects of sympathy on originality. *Thinking Skills and Creativity*, 21, 132–43.

[R78] Yavuz S.U. , Bordegoni M., Carulli M. (2018). A design practice on communicating emotions through sensory signals. *Concurrent Engineering*, 26, 147–56.

[R79] Yoshino A. , Okamoto Y., Doi M., et al. (2017). Functional alterations of postcentral gyrus modulated by angry facial expressions during intraoral tactile stimuli in patients with burning mouth syndrome: a functional magnetic resonance imaging study. *Frontiers in Psychiatry*, 8, 224. 10.3389/fpsyt.2017.00224 PMC5681843 29163243

[R80] Zabelina D.L. (2018). Attention and creativity. In: Jung R.E., Vartanian O., editors. *The Cambridge Handbook of the Neuroscience of Creativity*, New York: Cambridge University Press, 161–79.

