# Supplementary material for: Transcranial direct current stimulation (tDCS) targeting the postcentral gyrus reduces malevolent creative ideation
Source: Soc Cogn Affect Neurosci. 2023 Mar 24;18(1):nsad019. doi: 10.1093/scan/nsad019 (PMC10077332; doi:10.1093/scan/nsad019)
Supplement: nsad019_Supp [file nsad019_supp.zip › scan-22-251-File006.docx]

Table S1. The inter-rater correlation coefficient (ICC) of creative performance in study 1.

| MCT | | | BCT | | |
| --- | --- | --- | --- | --- | --- |
|  | Pre-test | Post-test |  | Pre-test | Post-test |
| Originality | 0.86 | 0.72 | Originality | 0.78 | 0.61 |
| Malevolence | 0.87 | 0.64 | Benevolence | 0.62 | 0.64 |

Table S2. The inter-rater correlation coefficient (ICC) of creative performance in study 2.

| MCT | | | BCT | | |
| --- | --- | --- | --- | --- | --- |
|  | Pre-test | Post-test |  | Pre-test | Post-test |
| Originality | 0.90 | 0.74 | Originality | 0.81 | 0.71 |
| Malevolence | 0. 87 | 0.71 | Benevolence | 0.60 | 0.70 |
